# Human Noroviruses in Swine and Cattle

**DOI:** 10.3201/eid1308.070005

**Published:** 2007-08

**Authors:** Kirsten Mattison, Anu Shukla, Angela Cook, Frank Pollari, Robert Friendship, David Kelton, Sabah Bidawid, Jeffrey M. Farber

**Affiliations:** * Health Canada, Ottawa, Ontario, Canada; † Public Health Agency of Canada, Guelph, Ontario, Canada; ‡ University of Guelph, Guelph, Ontario, Canada

**Keywords:** Norovirus, gastroenteritis, zoonoses, food microbiology, research

## Abstract

Human noroviruses are the predominant cause of foodborne gastroenteritis worldwide. Strains of norovirus also exist that are uniquely associated with animals; their contribution to the incidence of human illness remains unclear. We tested animal fecal samples and identified GIII (bovine), GII.18 (swine), and GII.4 (human) norovirus sequences, demonstrating for the first time, to our knowledge, that GII.4-like strains can be present in livestock. In addition, we detected GII.4-like noroviral RNA from a retail meat sample. This finding highlights a possible route for indirect zoonotic transmission of noroviruses through the food chain.

## Abstract 2

Detection of GII.4 norovirus sequences in animal fecal samples and retail meats demonstrates that noroviruses may be transmitted zoonotically.

Noroviruses (NoVs) are a widespread cause of nonbacterial gastroenteritis (*1*,*2*). These agents are highly infectious, and person-to-person contact, fomites, and food have all been implicated in their spread (*3*). NoVs infect hosts of all ages and can cause very large outbreaks in closed settings. For example, observations from cruise ships demonstrate that infection has spread rapidly (*4*).

Since the *Norovirus* genus comprises viruses that infect humans, pigs, cattle, and mice, the possibility for zoonotic transmission of infection exists. In general, zoonotic transfer could occur either indirectly through the food chain or directly through animal contact. Transfer of animal virus to humans may produce an infection more serious than that traditionally associated with NoV (*5*). High-profile examples of zoonotic viruses transmitted to humans include the highly pathogenic avian influenza virus, known as H5N1 (*6*), and the severe acute respiratory syndrome (SARS) coronavirus (*7*). If NoVs have the potential for zoonotic transmission, monitoring emerging viral strains and tracking their virulence characteristics will be important to limit the potential for negative public health effects from any zoonotic transfer event.

NoVs are nonenveloped, polyadenylated, single-stranded, positive-sense RNA viruses of the family *Caliciviridae* (*8*). The noroviral genome is 7.3–7.7 kb in length and contains 3 open reading frames (ORFs) (*9*). ORF1 encodes a polyprotein that is autocatalytically cleaved to produce an N terminal protein, the nucleoside triphosphatase, a 3A-like protein, the viral VpG, a 3C-like protease, and the RNA-dependent RNA-polymerase (*10*,*11*). ORF2 encodes the major capsid protein (*12*), and ORF3 codes for a minor structural protein (*13*). NoV strains are currently classified according to alignment of the amino acid sequence for the major capsid protein. This classification system divides the known NoV into 5 genogroups (*14*). Within these genogroups, 31 genetic clusters have been defined (*14*,*15*). Genogroup GI, GII, and GIV viruses infect humans; GIII NoV infect cattle; and GV NoVs infect mice (*16*,*17*). To date, NoVs detected in naturally infected pigs belong to GII (*15*,*18*). The porcine and human viruses belong to different clusters within GII; porcine viruses are identified as GII.11, GII.18, and GII.19, and the human viruses make up the remaining 16 GII clusters (*15*,*18*).

Porcine NoV detected in Japan, the Netherlands, and the United States is closely related to the human NoV (*15*,*18*,*19*). Furthermore, human NoV can replicate and induce an immune response in gnotobiotic pigs (*20*), which indicates that swine could serve as a reservoir for human NoV. In addition, the genetic similarity between human and swine NoV strains raises the possibility that porcine-human recombinants could emerge during co-infection of the same host (*15*).

Cattle can also be infected with NoV; however, this strain is less closely related to the human NoV (*21*). Bovine NoVs produce recombinants in a similar manner as human NoVs (*22*). Thus, a cow co-infected with both bovine and human strains of NoV could conceivably produce a recombinant virus with altered virulence properties.

In this study, we tested fecal samples obtained from pig and dairy farms, as well as retail meat samples for the NoV genome. Swine (GII.18) and bovine (GIII) strains were detected in the swine and cattle samples, respectively. We also identified GII.4 (human) NoV sequences in both types of animal sample. This is the first report, to our knowledge, of GII.4-like NoV in animal fecal samples. Additionally, we found a GII.4-like strain of NoV associated with a retail raw pork sample.

These data suggest a potential mechanism for zoonotic transmission of NoV to humans through meat, dairy, or farm samples from infected pigs and cows. These findings also highlight the possibility that a recombinant swine/human or bovine/human NoV could emerge with altered tropism or virulence characteristics.

## Materials and Methods

Swine fecal samples (N = 120) were collected in 3 sets from 10 farms in Canada farms. In September and October 2005, 4 composite samples were collected from each farm: 3 of fresh pen manure and 1 of stored pit manure. This sampling program was repeated in November and December of 2005 and again in April and May 2006. The fecal samples were placed into sterile containers, shipped overnight on ice, and stored frozen. Bovine fecal samples (N = 179) were collected from May to October 2006 from 45 different dairy farms in Canada. In this sampling program, farms were sampled only once each; 4 composite samples were taken at each farm, 1 each from calf pens (fresh), milking cow pens (fresh), heifer pens (fresh), and storage pits (stored). The dairy cattle samples were also placed into sterile containers, shipped overnight on ice, and stored frozen. A total of 156 retail meat samples were purchased, 15 at a time, during January–March 2006, and 6 at a time during July–November 2006. The last purchase consisted of only 4 samples. Each purchase was equally divided between samples of raw chicken, beef, and pork, except for the last, which had 1 extra chicken sample.

Fecal samples were suspended in 0.9% NaCl (5% wt/vol), vortexed briefly, and clarified through a combination glass fiber/PVDF 0.22-μm filter (Millipore, Mississauga, Ontario, Canada). RNA was extracted from a 140-µL sample of the resulting filtrate by using the QIAamp viral RNA extraction kit according to the manufacturer’s recommendations (QIAGEN, Mississauga, Ontario, Canada). Total RNA was extracted from 25-g meat samples by using Tri reagent (Sigma, Oakville, Ontario, Canada) and Dynabeads coated with oligo-dT (Invitrogen, Burlington, Ontario, Canada) as described (*23*).

The RNA was used as a template for 1-step reverse-transcription–PCR (RT-PCR) using Monroe region B primers (*24*) or Ando region A primers (*25*) and the OneStep RT-PCR kit (QIAGEN). Amplicons of 213 bp and 123 bp were considered to be presumptively positive for region B and region A, respectively. They were gel purified by using a QIAquick gel extraction kit (QIAGEN) and sequenced on both strands by DNA Landmarks (St. Jean sur Richelieu, Québec, Canada). The primer sequences were removed, and the resulting 172 bp or 81 bp of sequence was aligned to NoV standard genotypes (G1.1 GenBank accession no. M87661, GI.2 no. L07418, GI.3 no. U04469, GI.4 no. AB042808, GI.6 no. AF093797, GII.1 no. U07611, GII.2 no. X81879, GII.3 no. U02030, GII.4 nos. X86557 and AY502023, GII.5 no. AF397156, GII.6 no. AB039776, GII.8 no. AB039780, GII.9 no. AY038599, GII.10 no. AF504671, GII.11 no. AB074893, GII.12 no. AB045603, GII.16 no. AY772730, GII.17 no. AY502009, GII.18 no. AY823305, G3.1 no. AJ011099, G3.2 no. AF097917, G4.1 no. AF414426, G5.1 no. AY228235 [*14*,*15*]) using ClustalW (*26*). The alignments were subjected to phylogenetic analysis with 2,000 replicates for bootstrapping by using the Seqboot, Dnadist (F84), Neighbor, and Consense programs, as implemented in the PHYLIP package (*27*). Phylogenetic trees were generated by using TreeView software (*28*).

## Results and Discussion

### NoV Detection in Swine Fecal Samples

A total of 120 swine fecal samples were tested for NoV RNA by using region B primers (*24*); of these, 30 were confirmed by sequence analysis to contain partial genomic sequence from NoV. Detecting NoV RNA from 25% of swine fecal samples tested in this study contrasts with the results of other surveys, in which the rates observed were 2% in US adult swine (*15*), 20% in US finisher pigs (older, heavier pigs being finished before slaughter) (*29*), and 0% in Venezuelan pigs (*30*). The different specificities of the primer sets used in the 4 studies likely account for the differences in virus recovery.

**Figure 1:**
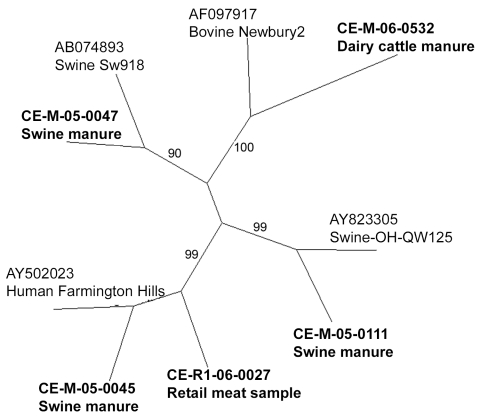
Unrooted neighbor-joining phylogenetic tree of representative noroviral strains and reference strains based on 172 bp of the RNA-dependent RNA polymerase region. GenBank accession nos. are indicated for the 5 reference strains (plain type), and the C-EnterNet sample codes are indicated for the representative strains identified in this study (**boldface type**). Bootstrap values are shown as percentages along the central branches

**Figure 2:**
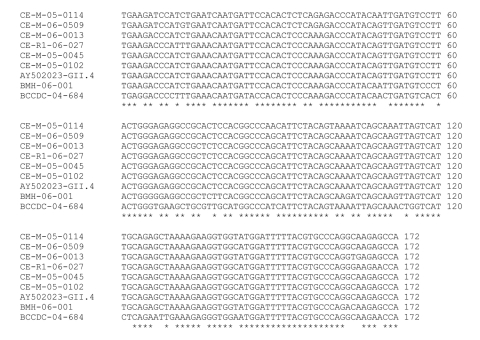
Nucleotide alignment of the 172-bp amplified region from the human GII.4-like strains. Four were detected in swine manure (CE-M-05–0114, CE-M-06–0013, CE-M-05–0045, and CE-M-05–0102), 1 in bovine manure (CE-M-06–0509), and 1 in a retail meat sample (CE-R1–06–027). The reference sequences provided are from the Farmington Hills reference strain (GenBank accession no. AY502023) and our laboratory strains, BMH-06–001 and BCCDC-04–684. Asterisks indicate identity at this position among all strains.

The sequence of the NoV polymerase region amplified was determined for the 30 strains and compared with those of reference strains. According to the region B sequence analysis, 3 different genotypes of NoV were detected in the Canadian pig samples (Figure 1), belonging to the swine GII.11 cluster (*18*), the swine GII.18 cluster (*15*), and the human GII.4 (Farmington Hills) cluster (*14*) (Figure 1). There were 22 strains of the swine GII.18 type, 6 of the swine GII.11 type, and 4 of the human GII.4 type; 2 samples contained both a swine GII.11 and a swine GII.18 virus. Sequences on both strands for the entire 172-bp region were obtained for 11 swine GII.18 viruses, 1 swine GII.11 virus, and 4 human GII.4 viruses. The 11 swine GII.18 strains had pairwise nucleotide identities ranging from 82% to 99% with no 2 identical strains (data not shown). Sequences from 2 of the 4 detected human strains were identical to each other and to the Farmington Hills reference strain (CE-M-05–0045 and CE-M-05–0102, Figure 2). The other 2 human NoV sequences amplified from swine manure were not identical to any other known sequences (CE-M-06–0013 and CE-M-05–0114, Figure 2). None of the amplicons sequenced were identical to the laboratory strains that we routinely use in our research activities (BMH-06–001 and BCCDC-04–684, Figure 2). Reasons for the incomplete sequencing of the remaining strains include low concentrations of RNA and overlapping peaks in portions of the chromatograms. A single representative of each group is shown in Figure 1 for clarity.

These results raise 2 possible issues related to public health. The first is that GII.4 NoV may be transferred directly from pigs to humans or from humans to pigs, providing a zoonotic source for human NoV outbreaks and a human source for pig NoV outbreaks. Second, pigs may be co-infected with both a human and a swine strain of NoV, potentially leading to recombination and generation of a new NoV strain with altered virulence properties. We did not identify GII.18-like and GII.4-like NoVs from the same manure sample; however, on 3 separate occasions, we detected both a GII.4-like and a GII.18-like NoV from the same farm on the same sampling date. A new variant of NoV with increased virulence has been seen in the GII.4 cluster in the past (*31*,*32*).

### NoV Detection in Bovine Fecal Samples

Of the 179 bovine fecal samples tested for NoV RNA with region B primers (*24*), 3 were confirmed by sequence analysis to contain NoV genomes. Thus, we detected NoV RNA in 1.6% of bovine fecal samples tested in this study, in contrast to other surveys, in which the rates were 72% of US veal calves (*33*) and 4% of Dutch dairy cattle specimens (*34*). This finding likely reflects the specificity of the primer sets used, although it may also relate to the type of animal tested.

The sequence for region B of the NoV polymerase was determined for these NoV strains and compared with reference strains. Based on this sequence, 2 different genotypes of NoV were detected in the Canadian dairy farm samples, belonging to the bovine GIII.2 Newbury cluster and to the human GII.4 Farmington Hills cluster. The complete 172-bp region B sequence was confirmed only for the sequence from CE-M-06–0509; this sequence was not identical to any of the other detected GII.4 sequences (Figure 2).

We also obtained an amplicon representing region A of the NoV polymerase from CE-M-06–0509. By region B phylogeny, the strain was classified as GII.4 (Farmington Hills) NoV (CE-M-06–0509; Figures 1, 2). Interestingly, the region A sequence did not share >77% identity with any of the NoV strains in the GenBank database. Phylogenetic comparisons to the standard viruses described by Zheng et al. (*14*) were not successful in assigning a genogroup and cluster for this sequence; bootstrap values were 16%–40% for most branches in the tree (data not shown). A large grouping of strains did emerge in this analysis, showing that in region A, this NoV strain was more closely related to GII.1, GII.2, GII.3, GII.4, GII.5, GII.10, GII.12, and GIII.1 sequences than to the other reference strains (data not shown). This finding indicates that the viruses detected and characterized as GII.4 NoV by using region B primers from swine and bovine manure (Figures 1 and 2) may represent novel NoV types, with genetic content slightly different from the previously recognized human, swine, and bovine-type species. This finding would also explain the difficulty we have had in obtaining region A, C, and D amplicons from the other manure samples (data not shown).

Human NoVs have not been shown experimentally to infect cattle; however, the human and bovine NoVs share a cross-reactive epitope in their capsid proteins (*35*). The risk for bovine NoV infection in humans is thought to be low (*21*). Co-infection of an animal with both human and bovine NoV may result in a recombinant agent with altered virulence, as the bovine NoVs have been shown to undergo extensive genetic recombination in a similar manner to the human viruses (*36*–*39*).

### NoV Detection in Retail Raw Meat Samples

To date, 156 retail meat samples have been processed and tested for the presence of NoV. One sample of raw pork has tested positive for a NoV of the GII.4 cluster (CE-R1–06–0027; Figures 1, 2). The region B sequence for this strain was not identical to any of the other sequences identified in this study (Figure 2). While we cannot determine the source of the contamination (i.e., at slaughter, during handling, or during packaging), our findings demonstrate that retail meat is a potential route for the indirect zoonotic transmission of NoV. This finding highlights the importance of proper handling and cooking for meat products. NoV titer is reduced by 4 orders of magnitude in <1 min at 71°C (*40*), the temperature which Health Canada recommends for the cooking of pork chops.

## Conclusion

This is the first report, to our knowledge, of GII.4-like NoV sequences in animal fecal samples. The GII.4 NoV sequences were all detected in fresh manure samples taken from animal pens, which limits the chances that they were derived from an unknown source of human waste. They were sampled from different farms on different days, and results from all processing and PCR control samples were negative. The use of the human-specific region B primer set (*24*) in our study allowed the identification of human NoV from animal specimens, in contrast to previous surveys,in which swine or bovine-specific RT-PCR primers were used (*15*,*18*,*19*,*34*).

Although GII.4 viruses can infect piglets experimentally (*20*), these data are the first indication that such infections may occur naturally as well. We have also identified partial GII.4 NoV genomic sequences for the first time in cattle feces. Retail samples have not been previously tested as part of a surveillance scheme for the presence of NoV. Our findings support previous suggestions that NoV may have a zoonotic route of transmission to humans (*15*). It follows that infected humans may pass the virus to livestock as well, and some outbreaks of gastroenteritis in farm animals may be prevented by controlling the access of ill workers to livestock. Our results also highlight the possibility that a recombinant swine/human or bovine/human NoV could emerge with altered tropism or virulence characteristics. In conclusion, we stress the importance of monitoring existing and emerging NoV strains to mitigate the potential impact of a recombinant NoV’s being transmitted to the human population from either a swine or a bovine source.
